# Knowledge structure and emerging trends on osteonecrosis of the femoral head: a bibliometric and visualized study

**DOI:** 10.1186/s13018-022-03068-7

**Published:** 2022-03-28

**Authors:** Haiyang Wu, Kunming Cheng, Linjian Tong, Yulin Wang, Weiguang Yang, Zhiming Sun

**Affiliations:** 1 Graduate School of Tianjin Medical University, No. 22 Qixiangtai Road, Tianjin, 300070 China; 2 Department of Intensive Care Unit, The Second Affiliated Hospital of Zhengzhou University, Zhengzhou, 450014 Henan China; 3 Department of Orthopaedic Surgery, Tianjin Huanhu Hospital, No 6, Jizhao Road, Jinnan District, Tianjin, 300350 China

**Keywords:** Osteonecrosis of the femoral head, Bibliometric analysis, Hotspots, VOSviewer, CiteSpace

## Abstract

**Background:**

Osteonecrosis of the femoral head (ONFH) is a common disabling disease with considerable social and economic impacts. Although extensive studies related to ONFH have been conducted in recent years, a specific bibliometric analysis on this topic has not yet been performed. Our study attempted to summarize the comprehensive knowledge map, development landscape, and future directions of ONFH research with the bibliometric approach.

**Methods:**

All publications concerning ONFH published from 2001 to 2020 were identified from Web of Science Core Collection. Key bibliometric indicators were calculated and evaluated using CiteSpace, VOSviewer, and the online bibliometric analysis platform.

**Results:**

A total of 2594 publications were included. Our analysis revealed a significant exponential growth trend in the annual number of publications over the past 20 years (*R*^2^ = 0.9663). China, the USA, and Japan were the major contributors both from the quality and quantity points of view. Correlation analysis indicated that there was a high positive correlation between the number of publications and gross domestic product (*r* = 0.774), and a moderate positive correlation between publications and demographic factor (*r* = 0.673). All keywords were categorized into four clusters including Cluster 1 (etiology and risk factors study); Cluster 2 (basic research and stem cell therapy); cluster 3 (hip-preserving study); and Cluster 4 (hip replacement study). Stem cell therapy-related research has been recognized as an important research hotspot in this field. Several topics including exosomes, autophagy, biomarkers, osteogenic differentiation, microRNAs, steroid-induced osteonecrosis, mesenchymal stem cells, double-blind, early-stage osteonecrosis, and asymptomatic osteonecrosis were considered as research focuses in the near future.

**Conclusion:**

Over the past two decades, increasing attention has been paid to global ONFH-related research. Our bibliometric findings provide valuable information for researchers to understand the basic knowledge structure, identify the current research hotspots, potential collaborators, and future research frontiers in this field.

## Introduction

Osteonecrosis of the femoral head (ONFH) is a common progressive disease typically characterized by reduction in vascular supply, bone metabolism disorder, and necrosis of the subchondral bone and eventually resulting in bone collapse of the femoral head [1]. It can be classified as traumatic and non-traumatic ONFH on the basis of diverse etiologies. Although the pathophysiology of this process has not yet been clearly elucidated, corticosteroid use, alcoholism, smoking, inherited coagulation disorders, as well as systemic lupus erythematosus are typically considered to be high-risk factors for non‐traumatic ONFH [1, 2]. In the USA alone, more than 20,000 new patients were diagnosed with non-traumatic ONFH each year, contributing to approximately 10% of the total number of total hip arthroplasties (THA) performed annually [3]. Another epidemiological study estimated that around 8.12 million Chinese population aged 15 years or over were affected by this condition, among which 55.75% of females and 26.35% of males have reported corticosteroid use [4]. Therefore, ONFH represent a major challenge in the orthopedic arena due to its high morbidity and disability rate, especially among young and middle-aged people.

Many tactics for treatment of ONFH depend on the severity of their condition, and the staging system formulated by the Association Research Circulation Osseous (ARCO) is one of the commonly used staging method in clinical practice [2]. According to the changes in the intraosseous blood supply in different phases of disease progression, the corresponding surgical and nonsurgical treatment strategies are recommended to prevent, or at least delay the progression toward the stage of femoral head collapse, in which THA is unavoidable [2, 5]. Despite the availability of numerous hip‐preserving surgical methods including core decompression, osteotomy, and bone graft, there still exists controversy regarding whether these treatment modalities are meaningful and valuable, as more than 80% patients with ONFH finally require THA [6, 7]. Additionally, some other controversies derived from ONFH, such as the pathogenesis, the optimal classification system, practicability of pharmacological treatments, optimal treatment protocols and surgical timing of THA, predictors of outcomes, and so on [2, 3]. Motivated by these concerns, ONFH have piqued the interest of researchers worldwide and a large number of related papers on this challenging topic have been published. To our knowledge, although some systematic reviews focusing on a specific subfield of ONFH research have been published, the global knowledge structure and research trends in this area have not been systematically studied yet.

Notably, the appearance of bibliometric method has compensated the shortage of literature reviews in a complementary fashion. Bibliometrics, first defined by Pritchard in 1969, is a visualization method to quantitatively assess the contribution of a research field by using mathematical and statistical approaches [8]. It is also regarded as an important approach to reveal the research trends and predict the research hotspots in a certain field [9, 10]. Over recent years, the application of bibliometric analysis is very extensive in the biomedical sciences, due to the explosion in the quantity of scientific publications and the availability of several freeware bibliometric tools [11, 12]. In the field of hip surgery, one recent study explored the global trends and hotspots of scientific research on femoroacetabular impingement from 2000 to 2019, based on 2471 originals articles indexed in Web of Science (WOS) [13]. Our research team and other groups have also investigated the publications on hip fracture [14], developmental dysplasia of the hip [15], and THA [16] by using bibliometric methods and assessed the co-authorship and co-citation network in these areas. However, to date, no studies have applied the bibliometric method to analyze the global research trends on ONFH. In view of this, the aims of this study were to (1) identify the current status of ONFH domain, including the distribution of annual outputs, the major players such as countries, institutions, and individuals; (2) analyze the cooperation networks at the level of countries, institutions, and authors; (3) summarize main research directions and hotspots; and (4) propose research frontiers and potential hotspots in the near future.

## Methods

### Source of bibliometric data and search strategies

Based on previous studies [14, 17, 18], the Science Citation Index Expanded (SCI-Expanded) of the Web of Science Core Collection (WOSCC) was selected as the main data source. The scientific literature was searched based on the titles (TI), abstracts (AB), and author keywords (AK) with the following search strategy: “femoral head necrosis” OR [(osteonecrosis OR necrosis) NEAR/2 (“femoral head”)] OR ONFH. The proximity operator of “NEAR/2” was used to combine search terms, which means two terms may have separated by a maximum of two words in any order (e.g., osteonecrosis NEAR/2 femoral head would have identified “osteonecrosis of the femoral head” and “osteonecrosis of femoral head”). A timespan of 20 years was set, and thus, only literature published from 2001 to 2020 was included. Publication language was restricted to English, and only original articles and reviews were eligible for this bibliometric analysis. All data utilized in this work were downloaded from public databases and, therefore, ethics committee approval or informed consent was not required.

### Data export and extraction

**Fig. 1:**
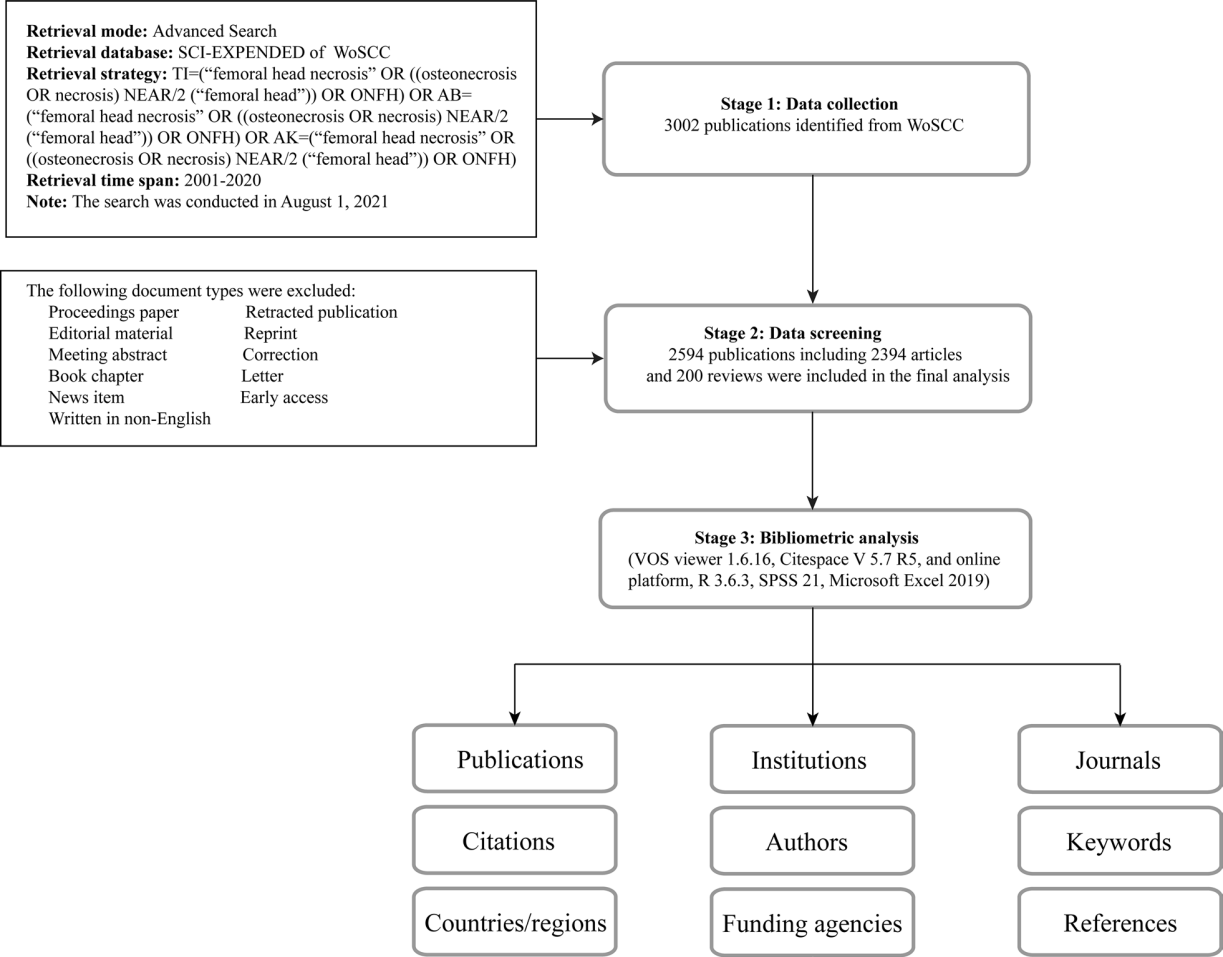
Flow diagram of the literature search and selection process

Considering that the database is regularly updated, all searches were done on a separate day to avoid this potential bias. By using the function of “export” in WoSCC, “full records and cited references” of retrieved records were exported as “tab delimited text (.txt)” to bibliometric tools for additional processing. Then the detailed data on the general information including annual publications, countries, institutions, authors, source journals, funding sources, research areas, number of citations, and Hirsch-index (H-index) were extracted. The above procedure was completed by two investigators independently, and any disagreements were solved through discussion or, if necessary, by the senior author. Moreover, journal impact factors (JIF) and quartile ranks were collected from the 2020 Journal Citation Reports. The detailed literature search and selection process are shown in Fig. 1.

### Bibliometric analysis

To obtain a more comprehensive analysis, three bibliometric tools, including an online platform and two software, were used to perform this study. First, the online bibliometric analysis platform (available at: https://bibliometric.com/) was used to conduct academic cooperation networks between countries. Then, VOSviewer 1.6.16 and Citespace V 5.7 R2 software were further used for mapping and visualizing bibliometric networks of scientific publications. VOSviewer, a freely available Java-based software developed by van Eck and Waltman at Erasmus University, is one of the frequently used bibliometric tools for quantitatively analyzing the academic literature [11]. In this study, VOSviewer was used to visualize the following network maps of ONFH research: network map of co-citation authors and journals; co-occurrence analysis of keywords. Specifically, co-citation network means that two items appear together in the bibliography of a third citing item, while co-occurrence network represents that the relationship of items is built according to the quantity of publications where they occur together [8, 11]. Generally speaking, the visualization maps mainly consist of nodes and links with different colors. Nodes in the visualization map represented the analyzed elements such as author, journal, or keyword, and the size of the nodes indicated the number of citations or occurrences [14]. The links between nodes reflected the relationship of co-citation or co-occurrence. An important parameter, total link strength (TLS), was used to quantitatively evaluate the strength of links [11, 14]. And the detailed descriptions of the maps could be found in the software manual at https://www.vosviewer.com/documentation.

Apart from that, we also employed another bibliometric software, called Citespace, which was developed by Professor Chaomei Chen of Drexel University, to perform further bibliometric analysis [12]. In the present study, CiteSpace was applied to conduct research cooperation relationships of authors and institutions; timeline view map of co-citation references; and references with the strongest citation bursts. CiteSpace is capable of generating different types of visualization map, such as the network map, the cluster view map, and timeline view map [12]. Overall, all these visualization maps are also comprised of nodes and lines representing different meanings. Betweenness centrality (BC) is an important indicator that could identify the relative importance of a node within the networks, and nodes with the highest BC value (≥ 0.1) are usually known as hubs nodes that usually marked with purple rings [17, 19]. More detailed software utilization skills and information about the visualization maps can be found in the CiteSpace manual (available at http://cluster.ischool.drexel.edu/~cchen/citespace/CiteSpaceManual.pdf).

### Statistical analyses

R software (v3.6.3.), SPSS (IBM SPSS Statistics 21, Inc., Chicago, IL, USA), and Microsoft Excel 2019 were used for descriptive analysis, statistical evaluation, data fitting, and plotting graphs. We computed the growth rate of publications over time with the following calculation formula as described previously by Guo et al. [20]. Pearson’s correlation coefficient test was used to assess the correlation between continuous variables, and correlations were considered significant when *p* value < 0.05.

## Results

### Publication outputs and trends

**Fig. 2:**
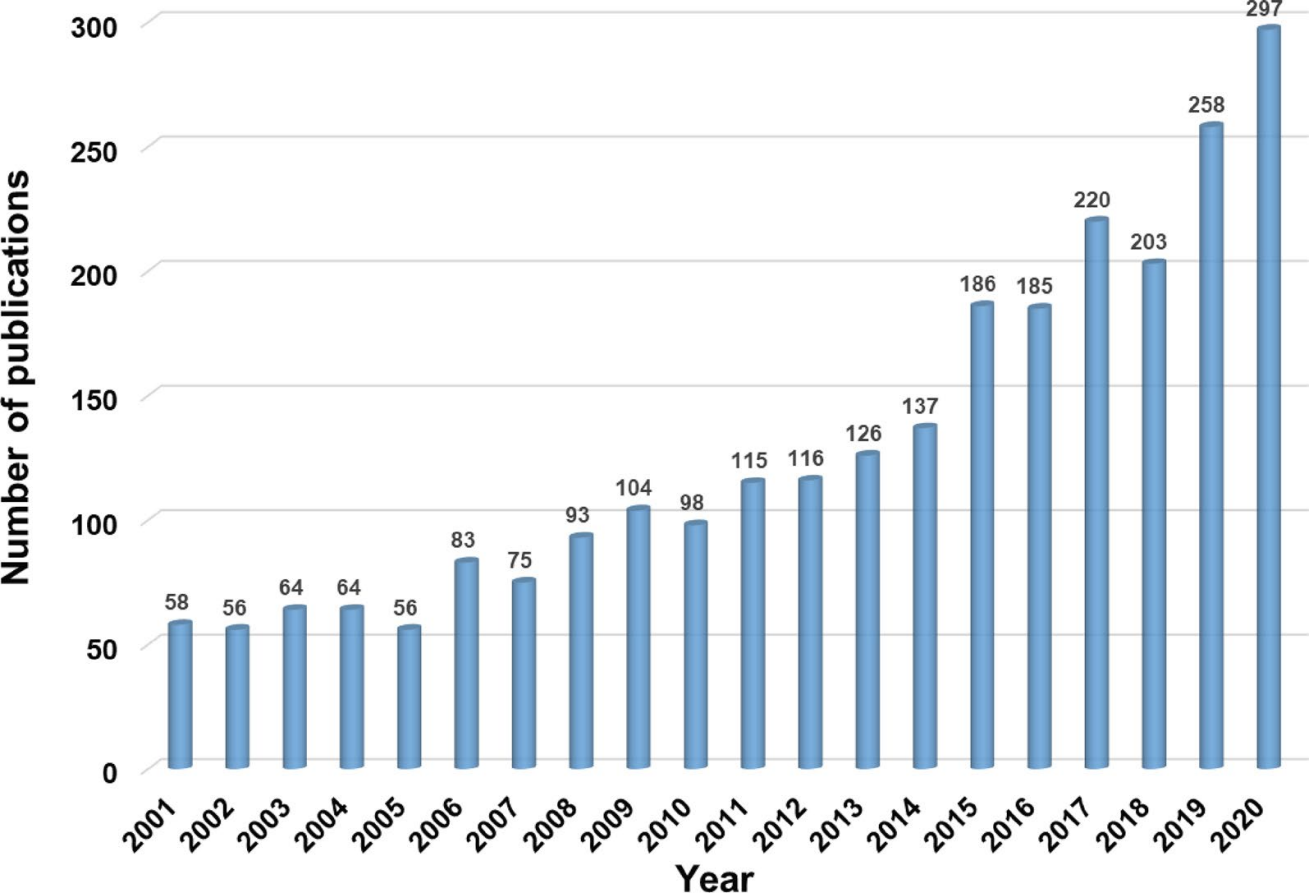
The specific numbers of annual documents regarding ONFH

Following the aforementioned screening strategy, a total of 2594 documents including 2394 original articles and 200 reviews related to ONFH were identified covering the period 2001–2020. Figure 2 presents the specific numbers of annual documents about ONFH. Model fitting curve revealed a significant exponential growth trend in the annual number of publications over the past 20 years (*y* = 44.943e^0.088*x*^, *R*^2^ = 0.9663). From 2001 to 2020, the average growth rate of scientific outputs was 33.41%.

### Analysis of countries/regions and funding agencies

**Fig. 3:**
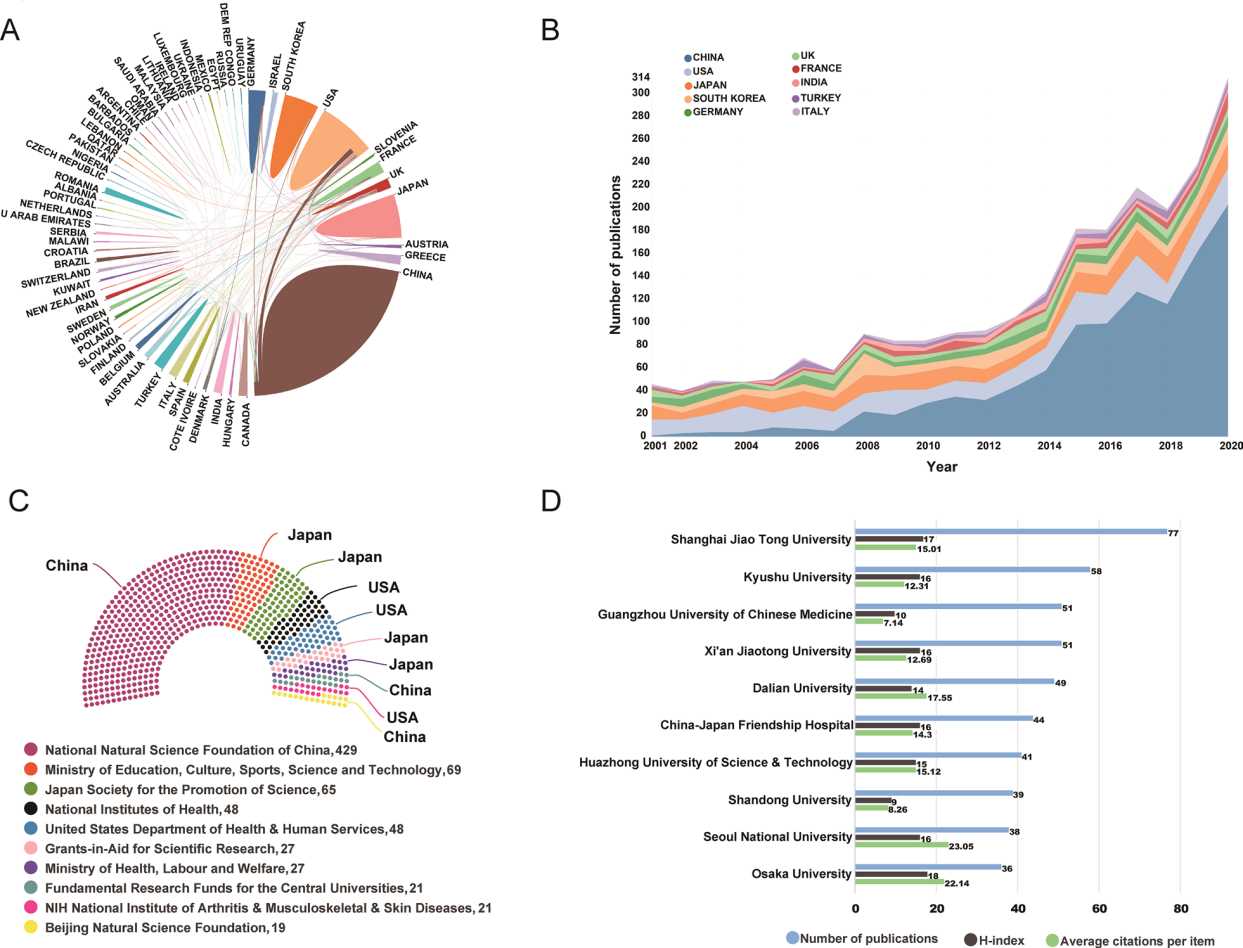
**A** International collaboration of countries in this domain. The area occupied per country is proportional to the number of documents. Line thickness reflects the closeness between countries, and a thicker line represents a stronger collaboration. **B** The annual number of publications in the top 10 prolific countries from 2001 to 2020. From 2010 onward, China has surpassed the USA for the first time and has remained that way since then. **C** The top 10 most active funding agencies in ONFH-related research. **D** The total number of publications, H-index, and average citations per item of top 10 most prolific countries in this field

All publications were distributed among 71 countries/regions. China had published the most publications with 1077 (41.52%) articles/reviews, followed by the USA [392 (15.11%)], Japan [278 (10.72%)], South Korea [167 (6.44%)], and Germany [123 (4.74%)] (Table 1). The results of correlation analysis indicated that there was a high positive correlation between the number of publications and gross domestic product (GDP) (*r* = 0.774, *p* < 0.001), and a moderate positive correlation was also found between publications and demographic factor (*r* = 0.673, *p* = 0.001). International collaboration of countries in this domain was also analyzed. As demonstrated in Fig. 3A, extensive collaboration was observed between productive countries. For instance, China collaborated closely with the USA, Australia, Germany, and South Korea. The USA, South Korea, Greece, Japan, and Italy have demonstrated active cooperation as well. In addition, the annual number of publications in the top 10 prolific countries from 2001 to 2020 is illustrated in Fig. 3B. Figure 3C lists the top 10 most active funding agencies in this field. Of these, four of them were from Japan, three from China, and the remaining three agencies were from the USA.

**Table 1:** Top 20 most productive countries related to ONFH research

Ranking	Countries	Publications, n	% of 2594	H-index	Average citations per document
1	China	1077	41.52	45	11.61
2	USA	392	15.11	55	29.35
3	Japan	278	10.72	33	16.28
4	South Korea	167	6.44	32	19.89
5	Germany	123	4.74	27	18.29
6	UK	102	3.93	28	24.54
7	France	73	2.81	23	25.14
8	India	59	2.27	15	12.63
9	Turkey	56	2.16	13	11.84
10	Italy	49	1.89	20	19.69
11	Greece	47	1.81	23	26.72
12	Canada	41	1.58	19	28.2
13	Switzerland	36	1.39	15	34.31
14	Belgium	31	1.20	18	36.13
15	Australia	27	1.04	12	27.52
16	Spain	27	1.04	10	11.85
17	Israel	24	0.93	12	38.13
18	Brazil	22	0.85	8	10.45
19	Austria	20	0.77	11	22.75
20	Iran	20	0.77	7	10.45

Ranking: according to the number of total publications

### Analysis of the most prolific institutions

**Fig. 4:**
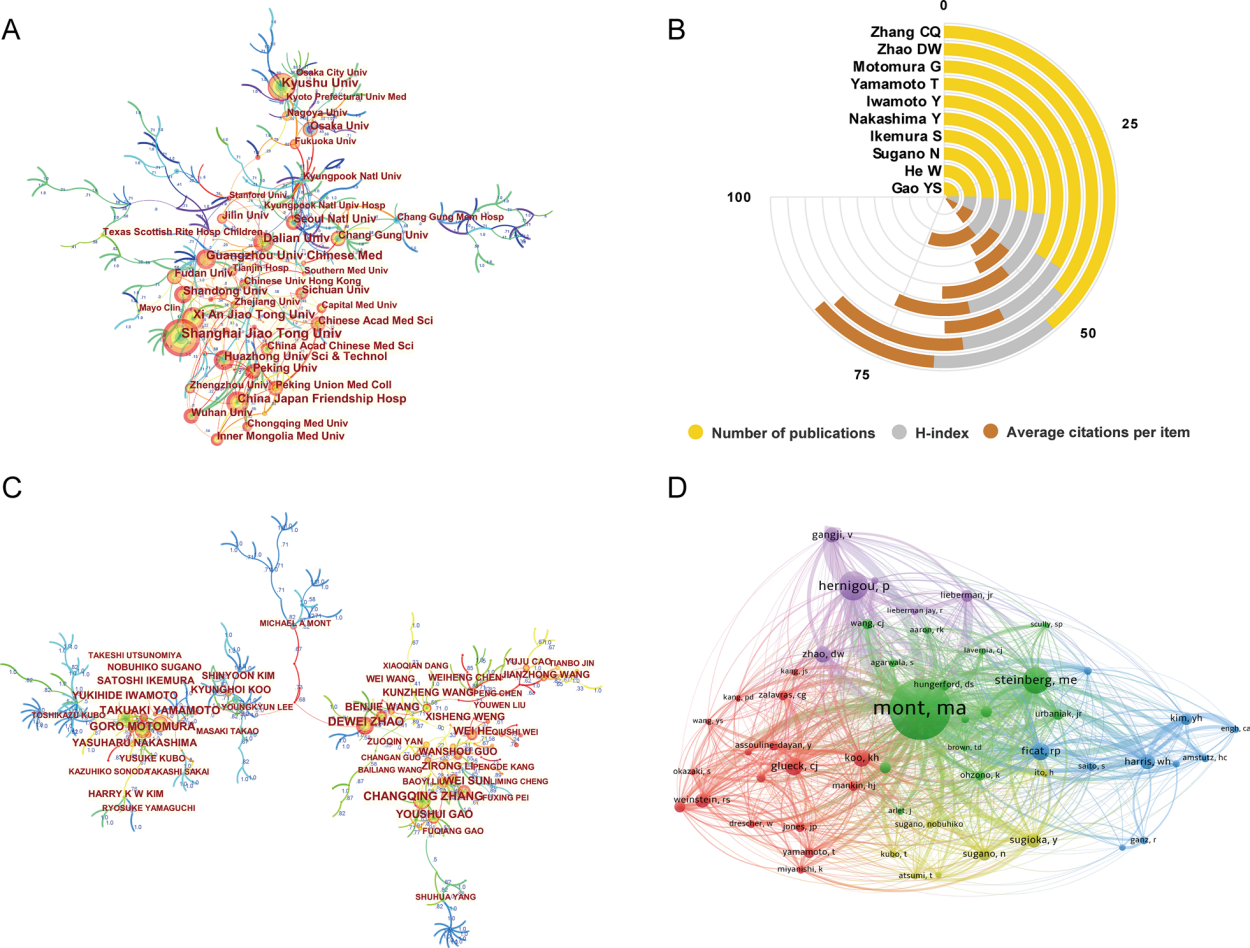
**A** The cooperation network map of institutions generated by CiteSpace. Each node represents an institution and the node size is proportional to the number of publications by that institution. The node with the highest BC value (≥ 0.1) are usually known as hubs nodes that usually marked with purple rings. The connecting line between nodes indicates the cooperation relationship, and the value of link strength is also displayed between lines. **B** The total number of publications, H-index, and average citations per item of top 10 productive authors in this field. **C** The cooperation network map of productive authors generated by CiteSpace. The graphical explanations are the same as in **A**. **D** Authors co-citation analysis by VOSviewer. Each node represents a different author, and the node size is proportional to the quantity of citations. The thickness of the connecting line between nodes indicates link strength of a co-citation relationship, which could be weighted by a quantitative indicator that is TLS

The top 10 most prolific institutions were laid out in Fig. 3D. All these institutions were from Asian institutions including 7 Chinese institutions, 2 Japanese institutions, and 1 Korean institution. Among them, Shanghai Jiao Tong University had the largest number of publications (77), followed by Kyushu University (58), Guangzhou University of Chinese Medicine (51), and Xi'an Jiaotong University (51). As for the other parameters, H-Index for Osaka University exhibited the highest value (18), followed closely by Shanghai Jiao Tong University (17). And Seoul National University had the highest value of average number of citations (23.05). A network visualization map of institution cooperation was generated by CiteSpace and illustrated in Fig. 4A.

### Analysis of the most influential authors

As shown in Fig. 4B, Zhang CQ from Shanghai Jiao Tong University contributed the highest number of papers, followed by Zhao DW from Dalian University, and Motomura G from Kyushu University. Figure 4C illustrates the cooperation network map of authors, none of the included authors had a BC value of more than 0.1. In addition, the co-citation network among authors was conducted using VOSviewer. As displayed in Fig. 4D, only authors with a minimum of 100 citations were included. There were 55 nodes, 5 clusters, and 1439 links in the network map. Among them, Mont MA from Sinai Hospital of Baltimore has occupied maximum node with the largest citations and TLS.

### Analysis of the higher-impact journals

**Fig. 5:**
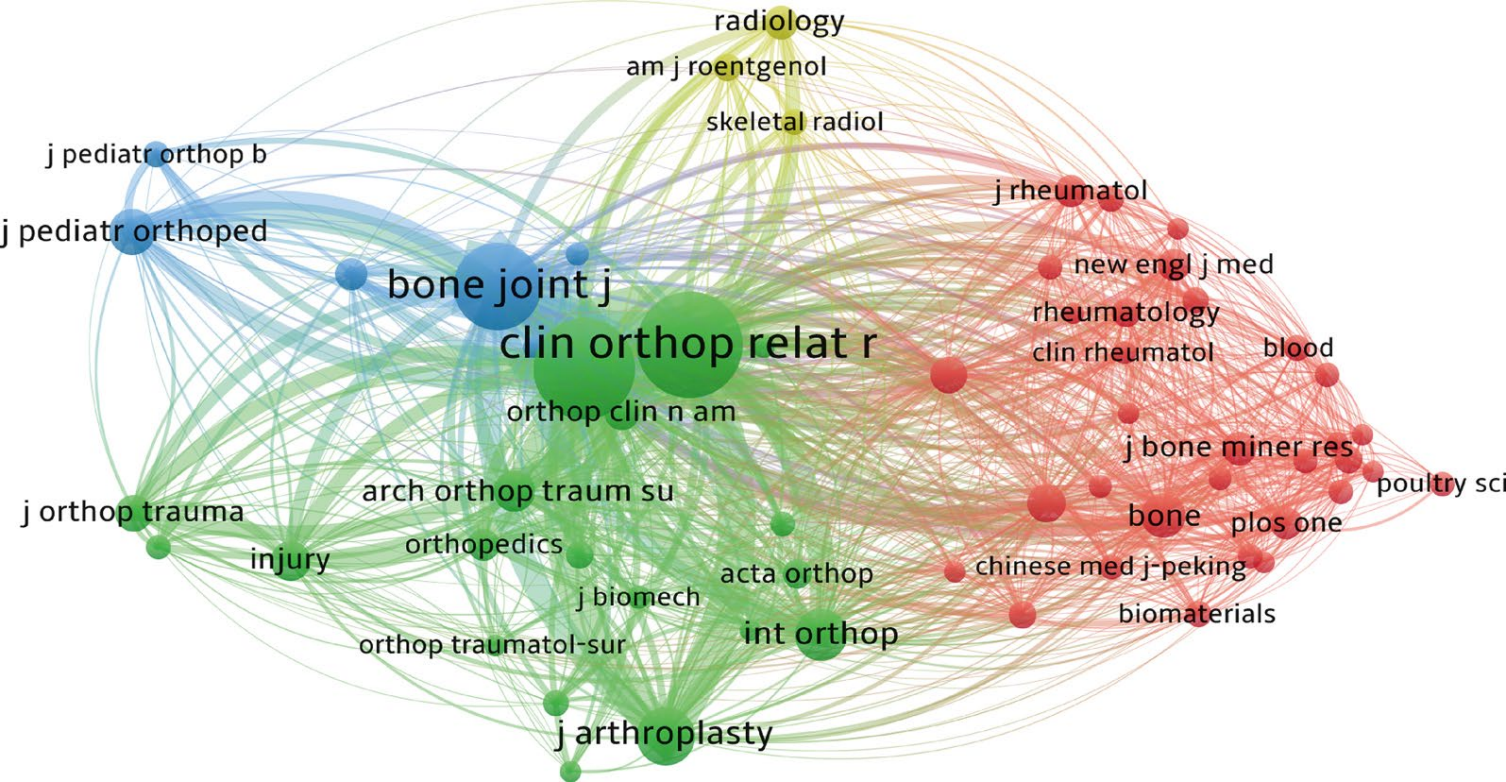
The network visualization map of journal co-citation analysis by VOSviewer. Each node represents a different journal, and the node size is proportional to the quantity of citations. Other graphical explanations are the same as in Fig. 4D

The top 10 most prolific journals were listed in Table 2. *International Orthopaedics* (JIF 3.075) has published the greatest number of 123 papers, followed by *Clinical Orthopaedics and Related Research* (JIF 4.291), and *Journal of Arthroplasty* (JIF 4.757), with 103 and 81 publications, respectively. According to JIF, JCR splits journals belonging to the same discipline into four equal parts, of the top 25% classified as Q1 and the top 25–50% being Q2, and so on. More than half of the top 10 journals were categorized in Q1 or Q2. Figure 5 shows the network visualization map of journal co-citation analysis. Only journals with more than 200 citations were depicted. Of the 60 journals satisfying the criteria, the top 5 co-cited journals were *Clinical Orthopaedics and Related Research*, *Journal of Bone and Joint Surgery American Volume*, *Bone & Joint Journal*, *Journal of Arthroplasty*, and *International Orthopaedics.*

**Table 2:** Top 10 journals with most publications in the field of ONFH research

Ranking	Journal title	Output	% of 2594	JIF (2020)	Quartile in category (2020)
1	International Orthopaedics	123	4.74	3.075	Q2
2	Clinical Orthopaedics and Related Research	103	3.97	4.291	Q1
3	Journal of Arthroplasty	81	3.12	4.757	Q1
4	Journal of Bone and Joint Surgery American Volume	79	3.05	5.284	Q2
5	Archives of Orthopaedic and Trauma Surgery	69	2.66	3.067	Q3
6	Bone & Joint Journal	67	2.58	5.082	Q1
7	Hip International	54	2.08	2.135	Q4
8	Medicine	53	2.04	1.889	Q3
9	BMC Musculoskeletal Disorders	50	1.93	2.355	Q3/Q4
10	Journal of Orthopaedic Surgery and Research	48	1.85	2.359	Q2

### Analysis of highly cited references

**Fig. 6:**
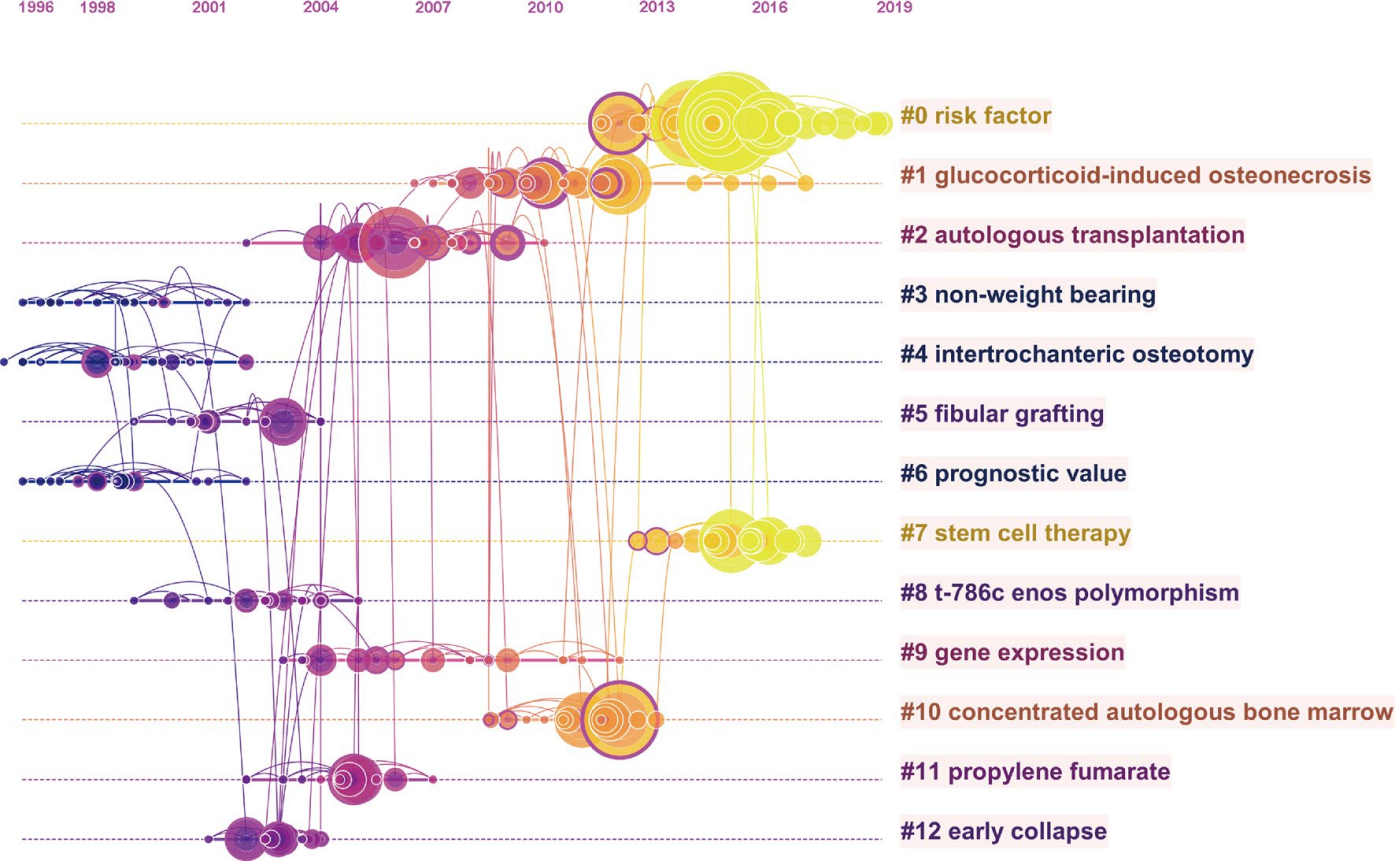
The timeline view map of reference co-citation analysis. For each cluster, the position of each node shows the time of publication of the document, and the node size represents the number of citations

**Fig. 7:**
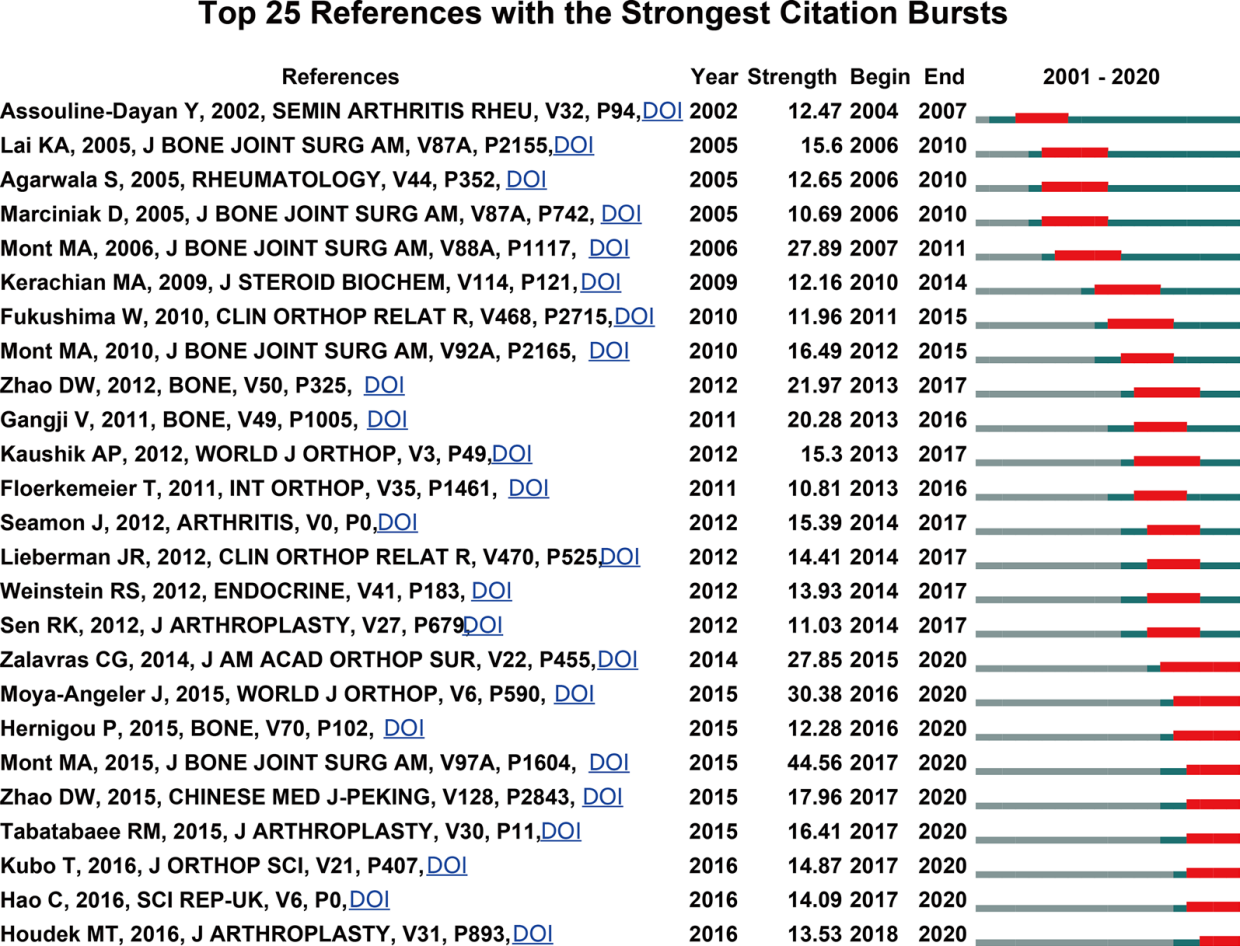
Top 25 references with the strongest citation burst in the ONFH field. The red segment represents the begin and end year of the burst duration

Reference co-citation analysis is one of the most attractive functions of CiteSpace, which is often applied to determine the research focuses on a given field. As shown in Fig. 6 and Table 3, all the nodes in the reference co-citation network map could be grouped into 13 major clusters. In CiteSpace, weighted mean silhouette value (S value) and the modularity value (Q value) are two indicators to evaluate the significance of clustering, and it is generally believed that *S* > 0.7 and *Q* > 0.3 represent the clusters are convincing. In this study, the mean value of S equals 0.7481 and that *Q* equals 0.7794, indicating the rationality of this clustering strategy. Moreover, as shown in Fig. 7, the top 25 references with the strongest citation bursts were identified in terms of their burst values.

**Table 3:** Main clusters of co-cited references

Cluster ID	Size	Silhouette	Label	Mean (year)
#0	32	0.965	Risk factor	2015
#1	31	0.9	Glucocorticoid-induced osteonecrosis	2010
#2	25	0.881	Autologous transplantation	2006
#3	21	0.932	Non-weight bearing	1998
#4	21	0.972	Intertrochanteric osteotomy	1998
#5	21	0.934	Fibular grafting	2001
#6	20	0.915	Prognostic value	1999
#7	19	0.893	Stem cell therapy	2015
#8	19	0.788	t-786c enos polymorphism	2002
#9	17	0.846	Gene expression	2007
#10	17	0.967	Concentrated autologous bone marrow	2010
#11	14	1	Propylene fumarate	2004
#12	14	0.951	Early collapse	2002

### Analysis of the most concerned keywords

**Fig. 8:**
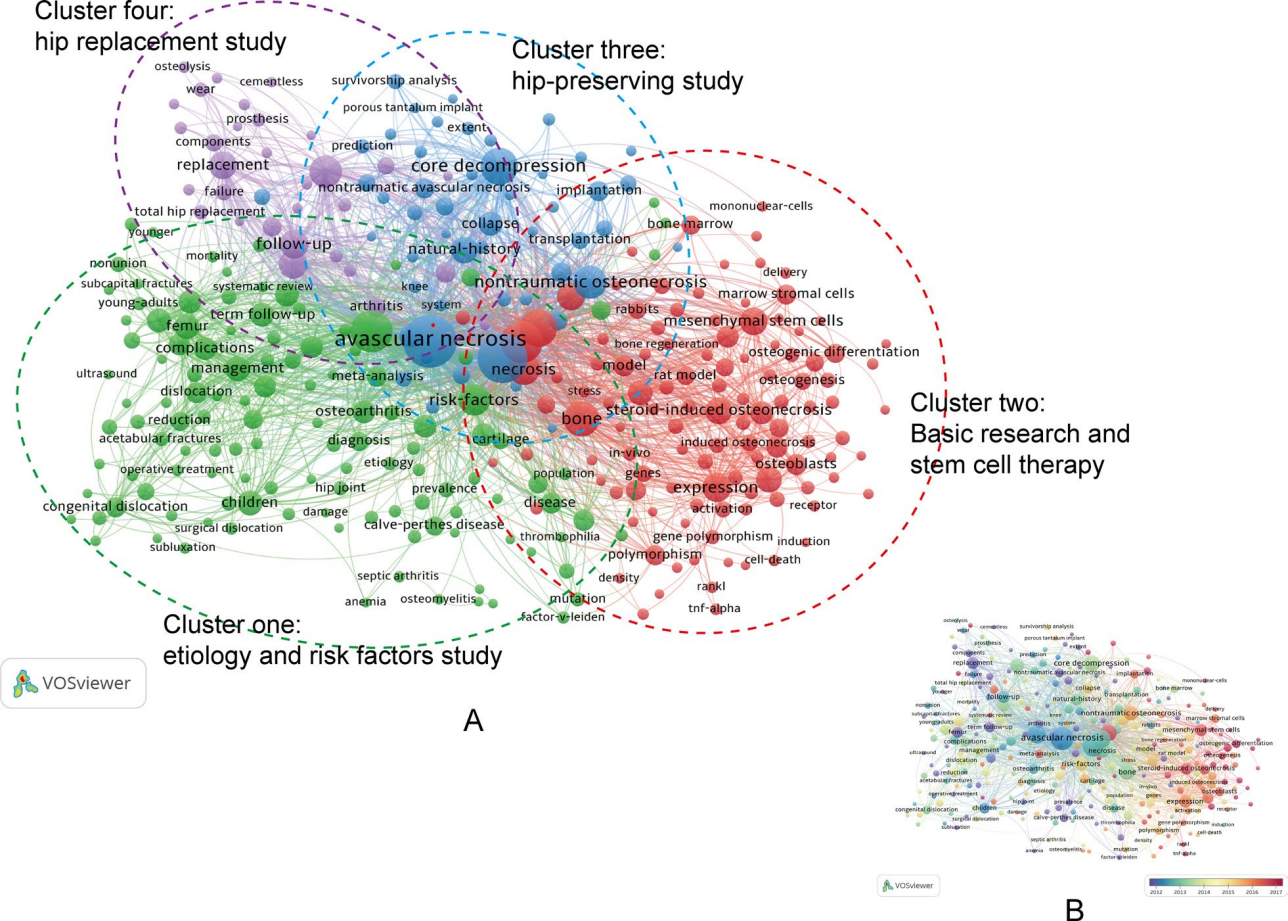
**A** Network visualization map of the co-occurrence network of keywords using VOSviewer. Each node represents a certain keyword. Nodes and font size represent the number of keyword occurrences. Keywords with close correlation will be assigned to one cluster with the same color. **B** Overlay visualization map of keywords analysis in the ONFH field. The color of each node shows the average appearing year (AAY) of the keyword, according to the color gradient shown at the bottom right. The blue-purple color reflected the keywords appeared relatively earlier, while the dark red nodes represented the recent occurrence

In this study, keywords that occurred at least 10 times were extracted from 2594 publications and analyzed by VOSviewer. After deleting meaningless keywords and merging keywords with the same meaning, a total of 319 keywords were identified. Based on the research categories of these keywords, VOSviewer software was able to divide all keywords into several major clusters with different colors. As shown in the network visualization map of Fig. 8A, all the included keywords were classified into the following four clusters: Cluster 1(etiology and risk factors study, green nodes); Cluster 2 (basic research and stem cell therapy, red nodes); cluster 3 (hip-preserving study, blue nodes); and Cluster 4 (hip replacement study, purple nodes). In addition to this, we also provided an overlay visualization map of keywords co-occurrence analysis in Fig. 8B.

## Discussion

### Worldwide research tendency of ONFH from 2001 to 2020

The number of publications in a certain field is able to reflect the productivity and development of the topic over the years. ONFH-related research has drawn increasing attention among scholars from 2001 to 2020. The global number of publications in this field has been gradually increased from 58 in 2001 to 297 in 2020, 71.05% of which was published in the last 10 years. One important reason for this growth was that the incidence of ONFH has been increasing worldwide and this devastating condition has become an increasingly prominent issue globally [1, 2, 21, 22]. Based on current trends, Mont et al. [23] reported that the total number of individuals affected by ONFH is estimated to reach 20 million by next 10 years worldwide. Besides, the increase in annual publications is inseparable from the advances in basic research and clinical trials in recent years [24, 25].

### Knowledge structure of ONFH-related publications

#### Countries

It is not difficult to see that the research centers of this field were mainly concentrated in East Asia, North America and Western Europe. The results of correlation analysis indicated that part of the discrepancy in the quantity of publications across different countries can be explained by factors of economy or population. The H-index is a bibliometric indicator that simultaneously measures the quality (mainly depends on citations) and quantity (number of documents) of publications in a journal, author, or country [19, 26]. In this study, the USA, China, and Japan were the top three countries with the highest H-index. Therefore, this result further proved that China, the USA, and Japan were the major contributors both from the quality and quantity points of view.

#### Institutions

In terms of research institutions, the most prolific institution was Shanghai Jiao Tong University, Kyushu University, Guangzhou University of Chinese Medicine, and Xi'an Jiaotong University. Nevertheless, inter-institutional cooperation levels were relatively low and primarily conducted in Asian research institutions. Furthermore, none of the institutions had BC value greater than 0.1, indicating that there was no one institution occupied the absolutely central position in the collaboration network [17, 19]. In view of this, strengthening cooperative networks in different research institutions or teams will be important for future studies whether for basic scientific researches or clinical trials.

#### Authors

An analysis of the most influential authors is helpful for scholars to learn existing partnerships and identify potential cooperative subjects at home and abroad. As shown in Fig. 4B, Zhang CQ, Zhao DW, and Motomura G were the top three contributors in this field. Zhang CQ and colleagues mainly focused on the application of free vascularized fibula grafting for the treatment of ONFH [27]. Apart from that, a study conducted by their research team, which reported the potential preventative effect of exosomes secreted by induced pluripotent stem cell-derived mesenchymal stem cells (iPS-MSC-Exos) on ONFH via promoting local angiogenesis, also received a great attention [28]. The co-citation analysis is usually considered to be a better method to evaluate the academic influence of a journal or a scholar [17]. As a result, the co-citation network among authors was conducted using VOSviewer. Mont MA from Sinai Hospital of Baltimore has occupied maximum node with the largest citations and TLS. Further analysis found that several high-quality reviews regarding diagnosis, classification systems, and treatment for ONFH, published by Mont et al. have achieved a high number of citations [29, 30].

#### Journals

As for journal analysis, *International Orthopaedics*, *Clinical Orthopaedics and Related Research*, and *Journal of Arthroplasty* were the top three journals with the most publications. Of the top 10 journals, although China is the largest publishing country, there is no one Chinese journal, indicating that China should strengthen several international journals in this field so as to attract more scientific publications and spread academic perspective. Notably, to address this issue, Chinese government has continuously increased its investment in the construction of first-class academic journals in recent years [31].

### An overview of research focuses and frontiers

#### Reference analysis

Reference co-citation analysis is often applied to determine the research focuses on a given field. All the publications and their references data were used to create homogeneous clusters, thus references that were connected tightly were divided into the same clusters, and conversely in different clusters. Our findings demonstrated that there were 13 major clusters in the co-citation network map. The largest cluster was “risk factor” (#0) [1, 2, 32]. Figure 6 shows the timeline view for the major clusters, which could illustrate the temporal and evolution characteristics of each cluster. The development of cluster 3, cluster 4, and cluster 6 occurred earliest, whereas cluster 0 (risk factor) and cluster 7 (stem cell therapy) were the recent research topics in the field of ONFH, which reflects the shift in research focus. Apart from that, burst detection of reference was another approach to track and capture the research hotspots. References with the strongest citation burst, indicating that they have received special attention during a period, are generally acknowledged as the research basics of frontiers in a certain field. As shown in Fig. 7, the strongest burst starting from 2015 was from the paper published by Mont MA and colleagues [30], followed by Moya-Angeler et al. [3] in 2015 and Mont MA et al. [29] in 2006. It also can be observed that reference with citation bursts first emerged in 2004, due to an article in 2002, and continued through 4 years. Of note, the burst of several references after 2015 is still ongoing, suggesting that these topics have gained considerable attention in recent years and deserve further attention for future periods of time. It is worth noting that most of these references involved stem cells therapy [33–35].

#### Keywords analysis

Generally, the author keywords of an article are usually the most representative terms used to give a brief overview of research theme, and the co-occurrence analysis of keywords is a common bibliometric method to present the knowledge content and structure visually and also uncover the evolution process and hot topics of a field [14]. Based on the research categories of these keywords, VOSviewer software was able to divide all keywords into several major clusters with different colors. As shown in the network visualization map of Fig. 8A, all the included keywords were classified into four main research clusters including Cluster 1(etiology and risk factors study); Cluster 2 (basic research and stem cell therapy); cluster 3 (hip-preserving study); Cluster 4 (hip replacement study). In addition to this, the VOSviewer software could impart all the included keywords with different colors based on their AAY. It can be seen that early research prior to 2015, the ONFH research was mainly focused on “hip replacement study” in cluster 4 and “hip-preserving study” in cluster 3, whereas keywords belong to in cluster 2 (“basic research and stem cell therapy”) had the relatively latest AAY than other clusters. Stem cells possess the ability to self-renew and differentiate into various cell types such as osteoblasts and endothelial cells to promote angiogenesis as well as bone regeneration [33]. In the meantime, they could also secrete a broad range of biological factors including multiple cytokines, growth factors, and exosomes to promote new blood vessel formation and rebuild blood supply in the necrotic regions [35]. As far as we know, multiple rigorous random controlled trials (RCTs) on the efficacy of stem cell therapy for early-stage ONFH have been initiated or currently in progress [23]. Yet at the same time, their clinical values remain to be further elucidated. Thus, stem cell therapy has become one of the most promising areas for ONFH.

Additionally, in cluster 2, these keywords with relatively latest AAY, such as exosomes, autophagy, biomarkers, osteogenic differentiation, microRNAs, steroid-induced osteonecrosis, and mesenchymal stem cells, may have great potential to be hot topics in the near future. For example, miRNAs are small non-coding RNAs that broadly regulate gene expression by specifically binding to complementary sequences in the 3’‐untranslated regions of their target RNAs. In recent years, the field of miRNAs has been emerged as a focus of ONFH research, and has received extensive attention from researchers in China and other countries [33]. Some scholars have used the microarray method to compare miRNA expression in patients with ONFH and in patients with femoral neck fracture. Of the 17 miRNAs identified with differential expression, 12 were up-regulated and 5 were down-regulated, suggesting that aberrant miRNAs expression might be involved in the pathogenesis of ONFH, and thus become diagnostic markers for ONFH [36]. Additionally, accumulating evidence demonstrated that multiple miRNAs could act as novel therapeutic targets for the prevention and treatment of ONFH by regulating osteogenic and adipogenic differentiation in MSCs [33, 35]. In terms of steroid-induced osteonecrosis, it is worth emphasizing that as the ongoing spread of coronavirus disease 2019 (COVID-19) globally, despite great strides in management, corticosteroids remain the mainstay for the treatment of moderate to severe acute respiratory syndrome (SARS), and with it arises challenges such as steroid-induced ONFH, especially in patients with the long-term or high doses use [37]. Some scholars have noted the potential risk and called for judicious use of corticosteroids in COVID-19 patients, particularly not recommended for routine use [38]. Aside from cluster 2, several topics with relatively latest AAY in other clusters including double-blind [39], early-stage osteonecrosis [40, 41], and asymptomatic osteonecrosis [42] also deserve further attention.

## Limitation

Despite the rigorous bibliometric analysis of this study, there were still several inevitable shortcomings. For example, we only analyzed bibliometric data from WOSCC database, which potentially missed several relevant publications reported in other databases, such as Scopus and PubMed [43]. Moreover, in consideration of only English publications in the study, it is unavoidable that several important publications in non-English language were omitted. As for the keyword clustering analysis, it might not be appropriate to combine the keywords with the same meaning into one node as different keywords might belong to clinical or basic research, respectively. And finally, the latest publications in 2021 were not incorporated since they lack sufficient time to accumulate considerable citations, which might in part affect our conclusions due to the rapid updating of research hotspots and frontiers.

## Conclusion

Overall, the ascending trend in the annual number of publications indicates that ONFH has attracted a great deal of interest from researchers worldwide, especially in the last 10 years. China, the USA, and Japan were the major contributors. And part of the discrepancy in the quantity of publications across different countries can be explained by the factors of economy or population. The most prolific institution was Shanghai Jiao Tong University. Professor Zhang CQ and Mont MA were the most influential authors with the highest number of publications and citations, respectively. According to keywords analysis, all the selected keywords could be categorized into four major clusters. Stem cell therapy-related research has been recognized as an important research hotspot in this field. It is recommended to pay more attention to these topics including exosomes, autophagy, biomarkers, osteogenic differentiation, microRNAs, steroid-induced osteonecrosis, mesenchymal stem cells, double-blind, early-stage osteonecrosis, and asymptomatic osteonecrosis, which have great potential to continue to be the research focuses in the near future.

## Data Availability

All the data can be downloaded from Web of Science Core Collection.

## Abbreviations

ONFH: Osteonecrosis of the femoral head
THA: Total hip arthroplasty
WOSCC: Web of Science Core Collection
GDP: Gross Domestic Product
JIF: Journal impact factors
TLS: Total link strength
BC: Betweenness centrality
AAY: Average appearing year
MSCs: Mesenchymal stem cells
